# Identification and characterization of a new variation in *DPM2* gene in two Chinese siblings with mild intellectual impairment

**DOI:** 10.3389/fgene.2023.930692

**Published:** 2023-04-19

**Authors:** Peiwei Zhao, Yanqiu Hu, Juan Hu, Cheng Li, Yufeng Huang, Lei Zhang, Sukun Luo, Hongmin Zhu, Jun Jiang, Xuelian He

**Affiliations:** ^1^ Precision Medical Center, Wuhan Children’s Hospital (Wuhan Maternal and Child Healthcare Hospital), Tongji Medical College, Huazhong University of Science and Technology, Wuhan, China; ^2^ Rehabilitation Department, Wuhan Children’s Hospital (Wuhan Maternal and Child Healthcare Hospital), Tongji Medical College, Huazhong University of Science and Technology, Wuhan, China; ^3^ Department of Neuroelectrophysiology, Wuhan Children’s Hospital (Wuhan Maternal and Child Healthcare Hospital), Tongji Medical College, Huazhong University of Science and Technology, Wuhan, China

**Keywords:** congenital disorders of glycosylation, DPM2, whole-exome sequencing, mutation, correlation of genotype–phenotype

## Abstract

**Introduction:** Congenital disorders of glycosylation (CDGs) are a genetically heterogeneous group of metabolic disorders caused by abnormal protein or lpid glycosylation. DPM2 is one subunit of a heterotrimeric complex for dolichol-phosphatemannose synthase (DPMS), a key enzyme in glycosylation, and only four patients with DPM2-CDG have been reported.

**Methods:** Whole-exome sequencing (WES) was performed in a Chinese family having two siblings with a mild form of DPM2-CDG with developmental delay, mild intellectual disability, hypotonia, and increased serum creatine kinase. Sanger sequencing was used to validate the variants identified in the siblings and their parents. In vitro functional study was performed.

**Results:** A homozygous mutation, c.197G>A (p.Gly66Glu) in exon 4 of DPM2 (NM_003863) was identified by whole exome sequencing (WES). In vitro functional analysis demonstrated that this variant increased the expression level of DPM2 protein and western blot revealed a significant decrease in ICAM1, a universal biomarker for hypoglycosylation in patients with CDG, suggesting abnormal N-linked glycosylation. We also reviewed the 4 previously reported patients carrying homozygous or compound heterozygous variants of DMP2 gene, and found that patients with variants within the region encoding the first domain had more severe clinical symptoms than those with variants within the second domain. However, the actual genotype-phenotype relationship needs more study.

**Discussion:** Overall, our study broadens the variant spectrum of DPM2 gene, attempts to explain the different phenotypes in patients with different DPM2 variants, and emphasizes the need of further functional studies to understand the underlying pathophysiology of the phenotypic heterogeneity.

## Introduction

Congenital disorders of glycosylation (CDGs) are a group of inherited metabolic disorders caused by abnormal glycosylation of proteins or lipids (Al Teneiji et al., 2017). Glycosylation is an essential and the most common cellular process for post-translational modification of proteins and lipids, functioning in cell–cell and macromolecular interactions, protein folding, and protein signaling (Moremen et al., 2012; Abu Bakar et al., 2018). Patients with glycosylation defects often present with a broad spectrum of complications, usually involving the nervous system, liver, heart, muscular, eyes, and immune system (Freeze et al., 2012; Rymen and Jaeken, 2014; Sadat et al., 2014).

Dolichol-phosphate-mannose (DPM) synthase catalyzes the formation of DPM and plays an important role in N-glycosylation, as well as in C-mannosylation, O-mannosylation, and the formation of glycosyl-phosphatidylinositol (GPI) anchors (Maeda and Kinoshita, 2008). DPM2, a hydrophobic protein with 84 amino acids, is a subunit of the heterotrimeric DPM synthase complex and is required for DPM enzyme activity, possibly by stabilizing the DPM3 protein (Maeda et al., 2000; Lefeber et al., 2009).

DPM2-CDG (MIM:615042) is caused by variants in *DPM2* gene and is an extremely rare CDG type. Until now, only four cases from three families have been reported, and three of them with the variant c.68A>G (p.Tyr23Cys) died before 3 years old due to severe epilepsy, muscular dystrophy, and acute respiratory infections (Barone et al., 2012; Radenkovic et al., 2021).

In this study, we reported two siblings from a Chinese family with dysmorphic features, developmental delay, mild intellectual disability, hypotonia, strabismus, and increased serum creatine kinase. A homozygous variant c.197G>A (p.Gly66Glu) was found in *DPM2* gene in these two patients. In addition, we reviewed all reported patients with DPM2-CDG and analyzed the correlation between genotypes and clinical presentations.

## Materials and methods

### Study subject

This study has been approved by the Institutional Review Board of Wuhan Children’s Hospital, Tongji Medical College, Huazhong University of Science and Technology (No. 2021R060-E01). Two siblings with developmental delay and mild intellectual disability were recruited in this study. Upon obtaining informed consent, peripheral venous blood was withdrawn from the siblings (at the age of 11 years and 19 years, respectively) and both parents. Genomic DNA was extracted from leukocytes of whole blood samples using the QIAamp Blood DNA mini kit (Qiagen, Hilden, Germany) according to the manufacturer’s instructions. RNA was extracted using TRIzol reagent (Invitrogen) if the peripheral venous blood was adequate. In this study, RNA was obtained from peripheral venous blood of the younger sister. Three healthy controls age-matched with the younger sister were recruited, and informed consents were obtained from all subjects' parents before peripheral venous blood samples were drawn from them.

### Whole-exome sequencing

Whole-exome sequencing (WES) and subsequent data analysis were conducted with the help of a third party medical laboratory (Chigene Lab, Beijing China). For WES, the target DNA fragments were enriched by hybridization to construct an exome library. High-throughput sequencing was performed using an Illumina NovaSeq 6000 sequencer. Single-nucleotide and indel variants were identified using the Genome Analysis Toolkit (GATK). Paired-end alignment was performed using the Burrows–Wheeler aligner (BWA). SNPs and indels were filtered and screened according to sequence depth and mutation quality. The variants were annotated using the OMIM, ClinVar, and HGMD databases. Candidate gene variants were confirmed by Sanger sequencing using self-designed primers in the patients and their parents. A protein sequence conservation analysis of mutation sites was conducted using MEGA software. The protocol used for WES was the same as described previously (Zhao et al., 2021).

### DPM2 plasmid construction and cell transfection

HCT116 cells were grown in DMEM supplemented with 10% fetal bovine serum (Gibco). The *DPM2* coding sequence was amplified from HCT116 cells with the oligonucleotides 5′-ATG GCC ACG GGG ACA GAC-3′ and 5′-TCA CTG AGC CTT CTT GGT CAC TCTC-3′ using Pfu DNA polymerase (TransGen Biotech). The wildtype DPM2 (WT-DPM2) construct was obtained by inserting the amplified fragment into the pcDNA3.1(+) expression plasmid using BamHI and EcoR I restrictions sites.

To generate *DPM2*-mutated protein (G66E-DPM2), site-directed mutagenesis was performed with the oligonucleotides 5′- GAC GCT CCT GTT TGT GGA ACT GTT CAT CTC -3′ and 5′- TCC ACA AAC AGG AGC AGC AGG AGG CCTG -3′ using overlap PCR. In addition, Y23C-DPM2 was generated as a control. All the positive clones were verified for the correct sequence by Sanger sequencing. HCT116 cells cultured in a 6-well plate were transfected with 1 μg plasmids using Lip3000 (Invitrogen) according to the manufacturer’s instructions. Proteins were then resolved by SDS-PAGE on 12% gels (Invitrogen) and electrotransferred onto PVDF membranes.

### *DPM2* expression analysis using real-time PCR

Total RNA was extracted from HCT116 cells using TRIzol reagent (Invitrogen, United States). The first complementary DNA was synthesized from RNA using reverse transcriptase (Takara, Dalian). Quantitative primers used were as follows: *DPM2*-F CTT GCC ATT CAT CGA CAG TCAG; *DPM2*-R CTT GGT CAC TCT CTT GGT CTTC.

Real-time PCR was performed using SYBR Green PCR kit (Takara, Dalian) and *GAPDH* as an internal control; the primers used for *GAPDH* were as follows: F: GAG CGA GAT CCC TCC AAA ATC AAG and R: GGT TCA CAC CCA TGA CGA ACATG.

### Western blotting

Cells were lysed in 1% NP-40 lysis buffer (50 mM Tris, pH 7.4, 150 mM NaCl, 1 mM EDTA, 1% NP-40, and 0.5% sodium deoxycholate) for 30 min on ice and then centrifuged at 12,000 rpm for 10 min. The protein concentration was determined by BCA assay (Thermo Fisher Scientific), and 20 ug total protein was separated by 12% SDS-PAGE and subsequently transferred to PVDF membranes. After blocking in 2% BSA (bovine serum albumin), membranes were probed with the following Abs: anti-Flag (200 ug/mL, Santa Cruz Biotechnology, Inc.), anti-ICAM1 (500 ug/mL, Proteintech, 60299-1-Ig), and anti-GAPDH (1000 ug/mL, Proteintech, 60004-1-Ig); all Abs were diluted (1:3000) during hybridization. Bound Abs were detected using secondary Abs (Cell Signaling Technology, Inc.) and enhanced chemiluminescence (ECL, Thermo Fisher Scientific).

### Immunofluorescence assay

To examine the localization of DPM2, a co-localization analysis of DPM2 with an endoplasmic reticulum (ER) marker (PDI) was performed. Cells adhered to poly-L-lysine-coated slides were fixed for 15 min with 3% paraformaldehyde and then permeabilized for 15 min in 0.1% Triton X-100/PBS (phosphate-buffered saline). After blocking for 60 min in 3% BSA/PBS, cell spots were incubated with the anti-DPM2 and anti-PDI antibody (Proteintech, 66422-1-Ig) for 60 min. Cells were washed in PBS three times and then incubated with goat anti-rabbit secondary Ab conjugated to Alexa Fluor 488 and Alexa Fluor 555 (Cell Signaling Technology, Inc.). Cells were then counterstained with DAPI, and fluorescent images were acquired on a confocal microscope (Leica Stellaris 5) using an oil immersion objective.

## Results

### Clinical report

The patient was admitted to the Rehabilitation Department in Wuhan Children’s Hospital due to developmental delay, mild intellectual disability, hypertonia, and strabismus at 11 years old. She was the younger daughter of healthy non-consanguineous parents without relevant family medical history. She was born at 40 weeks of gestation by caesarean section (on the request of her family) with a birth weight of 3.40 kg (50–75th) and a birth length of 50 cm (50–75th). Head circumference and Apgar scores were not available. She showed delayed motor milestones with head control at 4 months, turning over at 7 months, and unassisted walking at 3 years old. Even when she was 11 years old, she walked unsteadily, on tiptoe, with the center of gravity of the body in the sacrococcygeal region, and she had severe strephenopodia on the right. She had undergone Achilles tendon lengthening procedure. Now her walking posture improved, but she still needed the help of hands to get up or down stairs. Her upper limbs were normal and could freely move. Language development was also considerably delayed, and she spoke the first words at 4 years old. At present, she still had a poor expressive vocabulary with scant sentences and inadequate words and mild dysarthria. She also showed intellectual disability and was not able to count to 10. She had no obvious facial abnormalities other than strabismus which had not been corrected by surgery so far. In addition, she had recurrent infections at the age of preschool.

**FIGURE 1:**
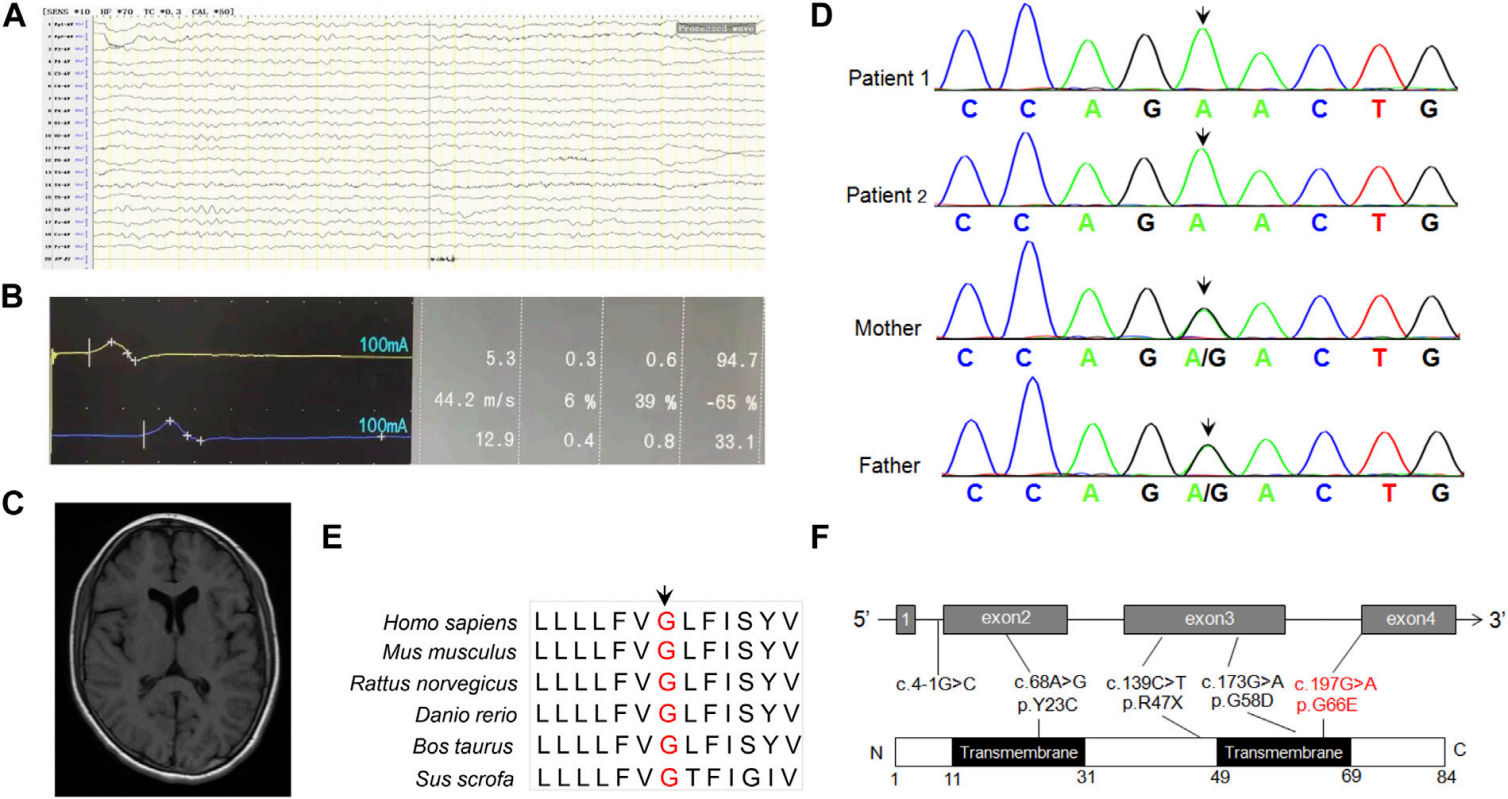
**(A)** EEG prompts slow background activity. **(B)** The motor nerve conduction amplitude of the right nervus peroneus communis was decreased, the terminal latency was prolonged, and the speed was normal, indicating that the axon and demyelination were damaged. **(C)** MRI shows abnormal signal foci in the white matter of the brain, and demyelination change in the younger sister. **(D)** Sanger sequencing of *DPM2* variants in the family of this study. **(E)** Conservation analysis of DPM2 protein among different species. The position of the variants at amino acid 66 is indicated in red and highly conserved throughout all indicated species. **(F)** Scheme of the distribution of *DPM2* variants. The variants in red were reported in the present study.

Blood biochemistry examinations showed normal parameters of liver function, including alanine transaminase (ALT), asparagine transaminase (AST), and gamma glutamyl transpeptidase (γ-GGT) but abnormal levels of creatine kinase (CK) (2097 U/L, reference: 20–250 U/L) and CK-MB (Muscle/Brain) (58 U/L, reference 0–25 U/L). There was no obvious abnormality in blood amino acid or urine organic acid analyzed by mass spectrometry. Electroencephalogram examination showed that occipital background activity was slightly slower. Electrophysiological evaluation showed slowed motor conduction velocity, longer motor latency, and decreased amplitude on the left median and ulnar nerves, but no sensory nerve action potential was detected in the median, ulnar, and sural nerves in the right lower limb, suggesting multiple peripheral nerve injury (Figures 1A, B). She did not undergo echocardiogram evaluation. The brain MRI scan showed slightly longer patchy signals on T1 and T2 and high intensity signals in the FLAIR (fluid-attenuated inversion recovery) sequence in the white matter of bilateral parietal lobes, an indication of demyelinating lesions (Figure 1C).

The elder sister had language and motor delay during childhood, and the developmental milestones were delayed. However, her conditions were better than those of her younger sister. She was able to control her head at 3 months, sat independently at 10 months, and walked unassisted at about 2 years old. Like her sister, she had the problem of recurrent infections at the age of preschool.

She had mild intellectual disability and exercise intolerance. Laboratory investigations showed increased creatine kinase (2022 U/L) and CK-MB (34 U/L) but normal liver and renal function parameters.

### WES analysis and putative pathogenic variant screening

In order to identify the pathogenic gene for a molecular diagnosis and prognosis, trio-WES (including the younger sister and parents) was conducted. Bioinformatics analysis was performed to identify candidate variants according to a filtering strategy based on population frequency, variant classification, and variant functional damaging prediction. A homozygous variant c.197G>A (p.Gly66Glu) in exon 4 of *DPM2* (NM_003863) was found in the younger sister, inherited from her heterozygous parents. This trio-WES result was confirmed by Sanger sequencing, which also identified the same homozygous variant in the elder sister (Figure 1D). The variant c.197G>A (p.Gly66Glu) was not found in any variant database (i.e., gnomAD, ClinVar, and 1000 Genomes). Bioinformatics analysis showed that this site is conserved among different species (Figure 1E), and the amino acid substitution is probably damaging, with a high PolyPhen-2 score of 1, a SIFT tolerance index of 0.006, a deleterious REVEL score of 0.837, and a damaging M-CAP score of 0.386. According to the standards and guidelines recommended by the American College of Medical Genetics and Genomics (ACMG), this variant should be classified as VUS (variant with uncertain significance). However, the grade could be upgraded to be likely pathogenic as more sources of evidence from the functional study were obtained (see the following paragraph) (PS3, *in vitro* or *in vivo* functional study supportive of a damaging effect on the gene or gene product; PM2, absent from controls or at extremely low frequency if recessive in the Exome Sequencing Project, 1000 Genomes Project, or Exome Aggregation Consortium; PP3, multiple lines of computational evidence support a deleterious effect on the gene or gene product). All germline variants (Tyr23Cys, c.4-1G>C, Arg47Ter, Gly58Asp) reported before, including the novel one identified in our study were shown in Figure 1F.

### DPM2 protein expression and subcellular localization

**FIGURE 2:**
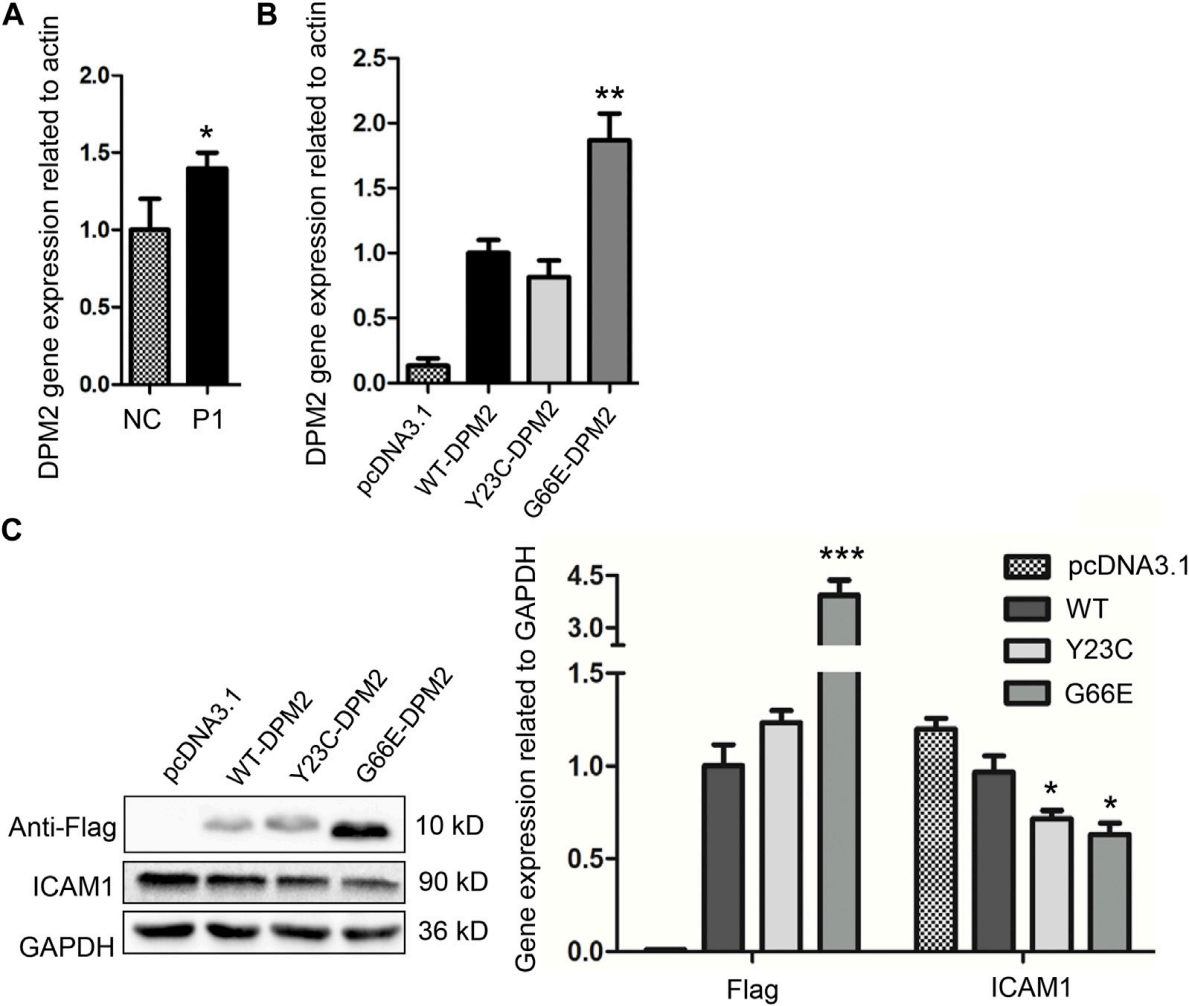
Expression level of *DPM2* in PBMCs of patient 1 and normal control **(A)**. **(B)** Expression level of *DPM2* mRNA in HCT116 cells transfected with wildtype or mutant *DPM2*. **(C)** Protein expression level of DPM2 and ICAM1 in HCT116 cells. NC, normal control; P1, patient 1 (*, *p* < 0.05; **, *p* < 0.01; ***, *p* < 0.001).

After performing quantitative real-time PCR by using RNA extracted from PBMCs of the younger sister and three age-matched normal controls, we observed that the *in vivo* level of *DPM2* mRNA was slightly but significantly higher (*p* < 0.05) in the younger sister than that of controls (Figure 2A), which could be due to individual variations. We then investigated the expression levels of Gly66Glu (G66E) variant and wildtype (WT), as well as a reported pathogenic variant Tyr23Cys (Y23C) in an *in vitro* system, by introducing plasmid expressing Flag-tagged G66E-DPM2, WT-DPM2, and Y23C-DPM2, into HCT116 cells, respectively. As shown in Figures 2B, C, expression of G66E-DPM2 dramatically increased at both mRNA and protein levels (*p* < 0.01 and *p* < 0.001, respectively), compared to WT-DPM2, whereas Y23C-DPM2 showed no significant changes, which could be due to the effect of the Gly66Glu variant. However, the efficiency of transfection was also an important factor that might be responsible for the observed changes.

**FIGURE 3:**
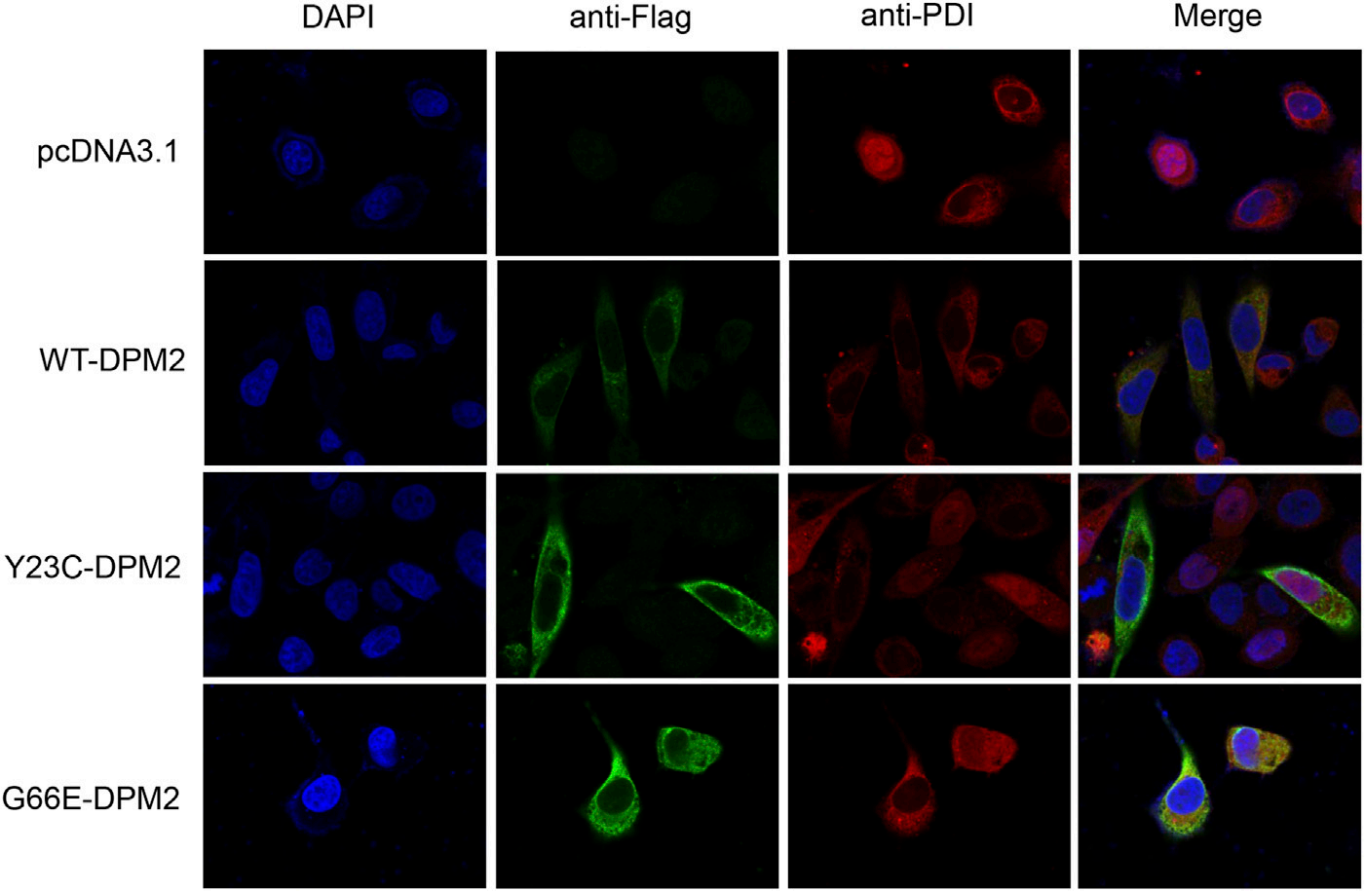
DPM2 protein expression and subcellular localization in HCT116 cells.

As reported previously, patients with Tyr23Cys DPM2 had severe clinical symptoms and died before 3 years old (Zhao et al., 2021), but this variant did not change the levels of mRNA or protein in our *in vitro* expression system (Figures 2B, C). In order to further figure out the possible pathogenic mechanism, immunofluorescence assay was used to examine the localization of mutated DPM2. However, neither Tyr23Cys nor Gly66Glu altered the cellular localization of DPM2 protein (Figure 3). Although the protein level and localization of Y23C-DPM2 were unchanged, ICAM1, a universal biomarker for hypoglycosylation in patients with CDG (He et al., 2012), was decreased in HCT116 cells (Figure 2C), which indicates that DPM2 may regulate glycosylation through a mechanism other than the protein level, consistent with reported involvement of Tyr23 in binding between DPM1 and DPM2 (Maeda and Kinoshita, 2008).

### Literature review of patients with DPM2-CDG

Literature review of DPM2-CDG was conducted by searching for all cases reported since 2012, the year in which DPM2-CDG was first reported (Zhao et al., 2021), using the keywords “*DPM2* gene” and “congenital disorders of glycosylation.” The searched databases included PubMed, Medline, and ClinVar. We reviewed two articles including four cases with DPM2-CDG (He et al., 2012; Zhao et al., 2021), three of which were reported by Rita Barone et al. in 2012. The demographic characteristics and clinical features of these patients (P3–P6), as well as the two siblings (P1–P2) we reported, are summarized in Table 1.

**TABLE 1:** Clinical characteristics of patients with DPM2-CDG.

Patient	Present study (P1)	Present study (P2)	P3 (10)	P4 (10)	P5 (10)	P6 (11)
**Sex**	F	F	F	M	M	M
**Age of onset**	11 Y	20 Y	After birth	After birth	After birth	23 Y
**Country**	China	China	Italy	Italy	Italy	India
**Year of publishing**			2012	2012	2012	2021
**Dysmorphic features**	Yes	Yes	Yes	Yes	Yes	No
**Microcephaly**	No	No	Yes	Yes	Yes	Yes
**Degree of developmental delay**	Mild	Mild	Severe	Severe	Severe	Mild to moderate
**Feeding difficulties**	No	No	Yes	Yes	Yes	No
						
**Muscle tone**	Hypertonia	No	Severe hypotonia	Severe hypotonia	Severe hypotonia	Hypotonia
**Seizure**	No	No	Treatment-resistant epilepsy	Treatment-resistant epilepsy	Treatment-resistant epilepsy	No
**Congenital heart disease**	No	No	No	No	No	Yes
**Hepatobiliary abnormalities**	No	No	Hepatomegaly and elevation of serum transaminases	NA	NA	No
**Skeleton**	Contracture of the ankle joint	No	No	Congenital contractures of the joints and scoliosis	Congenital contractures of the joints and scoliosis	
**Recurrent infection**	Yes	No	Yes	Yes	Yes	No
**Ophthalmological**	Strabismus	No	Optic atrophy	No visual tracking and strabismus	No visual tracking	
**Serum creatine kinase**	Elevated	Elevated	Elevated	Elevated	Elevated	Elevated
**EEG**	Abnormal EEG and background activity is slightly slow in the occipital area	NA	Bursts of paroxysmal multiple spikes	NA	NA	NA
**Brain MRI**	Abnormal signal of bilateral parietal white matter	NA	Loss of cerebral periventricular and subcortical white matter, without overt cerebellar atrophy	Mild cerebellar hypoplasia with severe vermis hypoplasia	NA	
**Outcome**	Alive, 11 years	Alive, 20 years	Died at 3 years	Died at 16 months	Died at 7 months	Alive, 20 years
**Allele 1**	c.197G>A (p. Gly66Glu)	c.197G>A (p. Gly66Glu)	c.68A>G (p. Tyr23Cys)	c.68A>G (p. Tyr23Cys)	c.68A>G (p. Tyr23Cys)	c.139C>T(p. Arg47Ter)
**Allele 2**	c.197G>A (p. Gly66Glu)	c.197G>A (p. Gly66Glu)	c.4-1G>C	c.68A>G (p. Tyr23Cys)	c.68A>G (p. Tyr23Cys)	c.173G>A(p. Gly58Asp)

As shown in Table 1, there were three male patients and three female patients, aged from 2 months to 23 years. The main clinical manifestations of the patients were progressive microcephaly, severe visual defect, intractable epilepsy, muscle damage, liver involvement, and decreased coagulation factors. These three patients reported by Rita Barone et al. in 2012 died at a very early age due to acute respiratory infections. The fourth patient was a 23-year-old adult male with truncal hypotonia, hypertonicity, congenital heart defects, intellectual disability, and generalized muscle wasting (He et al., 2012). In this study, we reported two cases of DPM2-CDG with much milder symptoms which broadened the phenotypic spectrum of the disease.

There are four germline variants (Tyr23Cys, c.4-1G>C, Arg47Ter, and Gly58Asp) reported previously. Three patients with Tyr23Cys and c.4-1G>C variants had severe symptoms, and one patient with compound heterozygous variants (Arg47Ter and Gly58Asp) had relatively mild symptoms. Two patients in this study carried the Gly66Glu variant, and their clinical presentations were much less severe than those of the previously described patients.

## Discussion

Glycosylation is an essential biological process for various protein or lipid modifications. Many human disorders of glycosylation pathways have now been identified, including defects in synthetic pathways for N-linked oligosaccharides, O-linked oligosaccharides, glycophosphatidylinositol (GPI) anchors, and dolichols (Moremen et al., 2012; Al Teneiji et al., 2017). In this study, we identified a novel variant in *DPM2* gene by WES in two Chinese siblings with mild developmental delay, hypotonia, and increased creatine kinase. Bioinformatics analysis and further *in vitro* experiment suggested that this variant was likely pathogenic.

DPM2 is one subunit of the heterotrimeric dolichol-phosphate-mannose (DPM) synthase complex (DPM1, DPM2, and DPM3), which catalyzes the synthesis of dolichol-P-mannose and plays a critical role in N-glycosylation as well as O-mannosylation (Barone et al., 2012). Deficiency in DPM1 has been associated with CDG syndrome due to deficient protein N-glycosylation, whereas defect in DPM3 causes a muscular dystrophy associated with abnormal O-mannosylation of dystroglycan (Waetzig et al., 2010; Barone et al., 2012; Yang et al., 2013; Bursle et al., 2017). DPM2 stabilizes the synthase complex (Watanabe et al., 2000). Although our study showed different expression levels between Gly66Glu and Tyr23Cys, the effect of transfection efficiency on protein expression should be considered. Furthermore, the tyrosine amino acid at 23 in the first transmembrane domain is highly conserved during evolution and is essential for proper function of DPM2 (Maeda et al., 1998). Thus, the protein expression level could not be a critical factor in maintaining the proper function of DPM2 in the process of glycosylation. Further study is needed to understand the detailed mechanism of DPM2 in modulating glycosylation and the effects of variants in *DPM2* gene on glycosylation.

DPM2-CDG is extremely rare, and only six cases from four families with five variants (c.139C>T, c.173G>A, c.197G>A, c.4-1G>C, and c.68A>G) have been identified (Table 1). DPM2 is composed of 84 amino acids and contains two putative transmembrane domains (amino acid residues 11–31 and 49–69, Figure 1F). One previous study showed that the introduction of two amino acid substitutions (Phe21 and Tyr23) to the first transmembrane domain of DPM2 abolished its ability to associate with DPM1, suggesting that the first domain of DPM2 is involved in association with DPM1 (Maeda et al., 1998). We also noticed that three patients with Tyr23Cys (homozygous or compound heterozygous) from two families had more severe phenotypes, including profound developmental delay, intractable epilepsy, severe hypotonia, and early fatal outcome (Barone et al., 2012) compared to other three patients with variants in the second transmembrane domain. However, more cases are needed to establish a genotype–phenotype correlation.

In addition, although electrophysiological evaluation did not find any significant muscle damage and muscle biopsy was not available, the younger sister had difficulties in movement, especially in going up and down stairs, the elder sister had exercise intolerance, and both had elevated creatine kinase. This evidence suggested that the siblings suffered from muscle damage. As mentioned previously, in addition to N-glycosylation, DPM synthase plays a critical role in O-mannosylation, the defect in which contributes to dystroglycanopathies, a subgroup of the congenital muscular dystrophies (van Tol et al., 2019). Therefore, it is possible that Gly66Glu affects the O-mannosylation by interfering with the function of DPM2, and more evidence is needed to support this hypothesis.

In conclusion, we identified and characterized a novel *DPM2* variant, c.197G>A (Gly66Glu) in two Chinese siblings with a milder form of CDG compared to patients with the Tyr23Cys variant. However, whether the phenotypes are linked to genotypes needs more study. Our study broadens the genetic spectrum of *DPM2* variants, emphasizing the importance of WES in assisting the diagnosis of rare diseases. Our study also raises questions with regard to the possible pathogenic mechanism which needs further study.

## Data Availability

The datasets for this article are not publicly available due to concerns regarding participant/patient anonymity. Requests to access the datasets should be directed to the corresponding authors.
